# Decline of FoxP3+ Regulatory CD4 T Cells in Peripheral Blood of Children Heavily Exposed to Malaria

**DOI:** 10.1371/journal.ppat.1005041

**Published:** 2015-07-16

**Authors:** Michelle J. Boyle, Prasanna Jagannathan, Lila A. Farrington, Ijeoma Eccles-James, Samuel Wamala, Tara I McIntyre, Hilary M. Vance, Katherine Bowen, Felistas Nankya, Ann Auma, Mayimuna Nalubega, Esther Sikyomu, Kate Naluwu, John Rek, Agaba Katureebe, Victor Bigira, James Kapisi, Jordan Tappero, Mary K Muhindo, Bryan Greenhouse, Emmanuel Arinaitwe, Grant Dorsey, Moses R. Kamya, Margaret E. Feeney

**Affiliations:** 1 Department of Medicine, University of California San Francisco, San Francisco, California, United States of America; 2 Center for Biomedical Research, The Burnet Institute, Melbourne, Australia; 3 Infectious Diseases Research Collaboration, Kampala, Uganda; 4 CDC, Atlanta, Georgia, United States of America; 5 Department of Medicine, Makerere University College of Health Sciences, Kampala, Uganda; 6 Department of Pediatrics, University of California San Francisco, San Francisco, California, United States of America; Imperial College, UNITED KINGDOM

## Abstract

FoxP3+ regulatory CD4 T cells (T_regs_) help to maintain the delicate balance between pathogen-specific immunity and immune-mediated pathology. Prior studies suggest that T_regs_ are induced by *P*. *falciparum* both *in vivo* and *in vitro*; however, the factors influencing T_reg_ homeostasis during acute and chronic infections, and their role in malaria immunopathogenesis, remain unclear. We assessed the frequency and phenotype of T_regs_ in well-characterized cohorts of children residing in a region of high malaria endemicity in Uganda. We found that both the frequency and absolute numbers of FoxP3+ T_regs_ in peripheral blood declined markedly with increasing prior malaria incidence. Longitudinal measurements confirmed that this decline occurred only among highly malaria-exposed children. The decline of T_regs_ from peripheral blood was accompanied by reduced *in vitro* induction of T_regs_ by parasite antigen and decreased expression of TNFR2 on T_regs_ among children who had intense prior exposure to malaria. While T_reg_ frequencies were not associated with protection from malaria, there was a trend toward reduced risk of symptomatic malaria once infected with *P*. *falciparum* among children with lower T_reg_ frequencies. These data demonstrate that chronic malaria exposure results in altered T_reg_ homeostasis, which may impact the development of antimalarial immunity in naturally exposed populations.

## Author Summary

In malaria endemic regions, immunity is slow to develop and does not provide substantial protection against reinfection. Rather, following repeated exposure, older children and adults eventually develop protection from most symptomatic manifestations of the infection. This may be due in part to the induction of immunoregulatory mechanisms by the *P*. *falciparum* parasite, such as FoxP3+ regulatory T cells (T_regs_). Prior human studies have shown that T_regs_ are induced by malaria parasites both *in vivo* and *in vitro*, but the role of these cells in immunity in children who are chronically exposed to malaria remains unclear. In this study, we assessed the frequency and features of T_regs_ among children from areas of high malaria transmission in Uganda. We found that this regulatory T cell population declined markedly with increasing malaria episodes. This loss was associated with decreased expression of TNFR2, which is a protein implicated in stability of T_regs._ Additionally, T cells from highly malaria exposed children demonstrated a reduced propensity to differentiate into T_regs_ following parasite stimulation. Together our data suggest that repeated episodes of malaria alter T_reg_ homeostasis, which may influence the development of immunity to malaria in children.

## Introduction

FoxP3+ regulatory CD4 T cells (T_regs_) play a central role in preventing autoimmunity and maintaining self-tolerance. In the setting of infection, T_regs_ help to maintain the delicate balance between pathogen-specific immunity and immune-mediated pathology. Preserving this equilibrium requires a complicated balance between regulatory and effector T cell activity. For instance, in the murine leishmania model, T_reg_-mediated suppression of effector immune responses interferes with complete parasite clearance—but paradoxically, the resulting pathogen persistence fosters the long-term maintenance of effector immune responses that are required for protection from reinfection [1,2]. Given their central role in immunoregulation, the timing, magnitude, and duration of T_reg_ activity must be fine-tuned for promote resolution of the effector immune response only after control of the pathogen has been achieved. Malaria, like many other parasite infections, has been reported to induce an expansion of the T_reg_ population [3]. However, the factors governing T_reg_ homeostasis in the setting of *P*. *falciparum* infection, which in high transmission regions is characterized by both recurrent symptomatic episodes in young children and persistent asymptomatic infection in older individuals, remain unclear, as does the role of T_regs_ in the immunopathogenesis of malaria.

*P*. *falciparum* infection in humans induces multiple immunoregulatory pathways that likely evolved to protect the host from severe malaria by down-modulating the acute inflammatory response, perhaps at the cost of interfering with clearance of parasitemia and development of immunologic memory. Several lines of evidence suggest that T_regs_ are induced during human *P*. *falciparum* infection and play a role in modulating the host response. Following experimental *P*. *falciparum* sporozoite infection of naïve human subjects, *FOXP3* mRNA is upregulated and peripheral blood CD25+CD4+ T cells expand [4]. In rural Gambia, the percentage and absolute count of FoxP3+CD127^low^ CD4 T cells were shown to increase following the malaria transmission season, and are significantly higher among malaria-exposed rural Gambians than among ethnically matched urban Gambians with no malaria exposure [5]. Moreover, a number of studies have shown that peripheral T_reg_ frequencies correlate with parasite burden in infected individuals [6–8]. Together these data suggest that T_regs_ are induced by *P*. *falciparum* infection *in vivo*. This conclusion is further supported by *in vitro* studies demonstrating that FoxP3+ T_regs_ are induced by co-culture of PBMC with *P*. *falciparum*-infected red blood cells or parasite schizont extracts [9–13].

Induction of T_regs_ by parasite antigens may have implications for the development of a host-protective immune response. *FOXP3* mRNA levels in children with acute malaria have been shown to correlate inversely with the magnitude of the subsequent Th1 memory response to *P*. *falciparum* measured 28 days after infection [6]. Similarly, *FOXP3* expression among malaria-naive adults following experimental sporozoite vaccination correlates inversely with the subsequent Th1 memory response [14]. It is possible that *P*. *falciparum* induction of T_regs_ may contribute to the failure of the adaptive immune response to mediate parasite clearance, as has been demonstrated in other parasitic infections such as leishmania and filariasis [1,2,15]. However, the role of T_regs_ in protection or risk from symptomatic malaria remains unclear. High frequencies of CD25^high^ T cells (putatively regulatory T cells) were associated with increased risk of malaria in one prospective cohort study [16]. Consistent with this, among previously naïve adults experimentally infected with malaria, T_reg_ induction was associated with increased parasite replication rates [4]. Further, a recent study in children and adults in Indonesian Papua found a trend towards lower proportions of activated T_regs_ in individuals who had asymptomatic infection compared to symptomatic malaria or healthy controls, suggesting dampened activation of T_regs_ may be associated with decreased risk of disease [17]. However, it has also been suggested that T_regs_ may serve a protective role in preventing immunopathology during infection [18,19]. Murine studies have failed to provide clear resolution of this issue, as different models have yielded conflicting data. Early reports described enhanced control of parasitemia and improved survival in mice experimentally depleted of T_regs_ [20], but subsequent studies that used more precise definitions of T_regs_, different depletion regimens, or different parasite strains have failed to demonstrate a consistent host-protective role (summarized in [19]).

To better understand the role of T_regs_ in the immunopathogenesis of malaria in the setting of chronic exposure, we assessed the frequencies and phenotypic features of T_regs_ in Ugandan children of varying ages and malaria exposure histories. Our results indicate that while T_reg_ frequencies are expanded in a high compared to low transmission settings, in high transmission settings children with repeated malaria infection experience a marked and progressive decline in peripheral blood T_regs_, accompanied by reduced *in vitro* induction of T_regs_ by parasite antigen and decreased expression of TNFR2. This loss of circulating T_regs_ may have implications for the development of protective immunity to malaria, and suggests that chronic antigen stimulation, such as that observed in areas of chronic *Plasmodium* infection, may result in pathogen-driven alteration of T_reg_ homeostasis.

## Results

### The frequency of FoxP3+ regulatory CD4 T cells in peripheral blood declines with increasing prior malaria exposure

**Fig 1:**
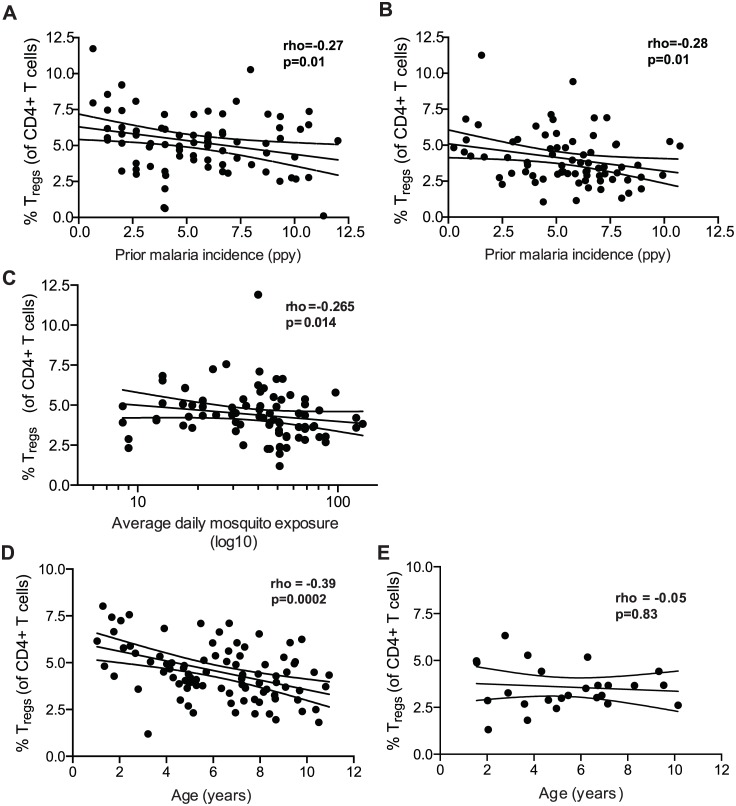
Regulatory T cells decline with increasing prior malaria incidence and mosquito exposure among children in a high transmission setting. Regulatory T cell frequencies were analyzed as the percentage of FoxP3+CD25+ of CD4+ T cells from **(A)** fresh whole blood in 2-year old (PROMOTE-cohort, no chemoprevention control arm) and **(B)** and frozen PBMCs from 4-year olds (TCC) and the association with prior malaria incidence analyzed. In both 2 and 4 year olds, T_reg_ frequencies declined with increasing prior malaria incidence. **(C/D)** Regulatory T cell frequencies, analyzed as the percentage of FoxP3+CD25+CD127^dim^ of CD4+ T cells from 1 to 11 year old children (PRISM cohort, high transmission Nagongera, Tororo District), declined with increasing mean daily household mosquito exposure (from monthly CDC light traps) **(C)** and age **(D)**. **(E)** The relationship between T_reg_ frequencies and age was analyzed in children from the low transmission Jinja District; there was no decline in T_reg_ frequencies with age in children from the low malaria transmission settings. For all analyses, Spearman’s rho and p are indicated.

To investigate the relationship between T_reg_ frequencies and prior malaria exposure, we measured peripheral blood T_reg_ frequencies in 2 separate cohorts of children in the high malaria transmission region of Tororo District, Uganda (annual entomological inoculation rate (aEIR) 310 bites ppy [21]). In both cohorts, participants were followed prospectively from enrollment at approximately 6 months of age, with comprehensive documentation of all malaria episodes at a dedicated study clinic, and at the time of analysis were either 2 years old (PROMOTE cohort, no chemoprevention control arm, n = 82) or 4 years old (TCC cohort, n = 75) (S1 Table). T_reg_ frequencies were measured as the percentage of CD4 T cells that were FoxP3+CD25+ (for gating strategy see S1 Fig). Within both the 2-year-old and 4-year-old cohorts, there was a strong inverse relationship between T_reg_ frequencies and prior malaria incidence (Spearman’s r = -0.27, p = 0.01, and r = -0.28, p = 0.01 respectively; Fig 1A and 1B). This inverse relationship was strengthened by further gating on the CD127^dim^ subset, which more stringently defines suppressive T_regs_ (Spearman’s r = -0.36, p = 0.001; assessed in 4-year-old cohort only, for gating strategy see S1B Fig). The frequency of T_regs_ among children who had asymptomatic *P*. *falciparum* infection at the time of assessment (determined by blood smear) did not differ from that of uninfected children (Wilcoxon ranksum p = 0.951). Furthermore, there was no relationship between the frequency of T_regs_ and the duration of time since the last malaria episode, which might be expected if T_regs_ transiently increase in response to acute malaria (Spearman’s r = 0.094, p = 0.422), similar to what has been shown in malaria-naïve adults [4]. We measured CD4 T cell responses to *P*. *falciparum*-infected red blood cells from blood samples obtained concurrently (TCC cohort, n = 56), but we observed no statistical relationship between the frequency of T_regs_ and other effector or regulatory T cell populations, including cells producing IFNγ (p = 0.65), TNFα (p = 0.17), or the recently described IL10-producing “self-regulatory” CD4 T cells (p = 0.99) [22–25].

The cross-sectional data above are consistent with either a malaria-driven decline in peripheral T_reg_ frequencies or an increased susceptibility to symptomatic malaria among children whose T_reg_ frequencies are inherently low. To distinguish between these possibilities, we measured T_reg_ frequencies in a third cohort of children residing in the same high transmission Nagongera, Tororo District (PRISM cohort, age 1 to 11 years, n = 91 [21]), in whom mosquito exposure was directly measured using CDC light traps within the homes of individual cohort participants [26]. In this cohort, we observed an inverse relationship between T_reg_ frequencies and mean daily household mosquito exposure, consistent with a parasite-driven decline in T_regs_ (Spearman’s rho = -0.265, p = 0.014, Fig 1C). In contrast to the younger cohorts of children described above, we did not observe an inverse correlation between T_regs_ and the incidence of prior clinical malaria in this cohort (Spearman’s rho = 0.043, p = 0.685), likely because older children do not develop symptomatic clinical malaria with each *P*. *falciparum* infection, and thus malaria incidence is not a good measure of total *P*. *falciparum* exposure beyond early childhood. There was, however, a strong inverse relationship between T_regs_ and age (Spearman’s rho = -0.385, p = 0.0002; Fig 1D), suggesting that T_regs_ progressively decline with age in this high endemnicity setting. This decline was not attributable to age-related changes in total lymphocyte counts, as a similar relationship was observed when absolute numbers of T_regs_ (per μl of blood) were calculated by normalization to absolute CD4 cell counts in a subset of children (r = -0.424, p = 0.025; n = 28, S2 Fig). To investigate whether the age-related decline in T_reg_ frequencies was unique to this high malaria transmission setting, we compared T_reg_ frequencies among children age 1.5 to 11 years who were enrolled in the observational malaria cohort (PRISM), but at the low transmission Jinja District (aEIR 2.8 bites ppy [21]). Among children at the lower transmission site, T_reg_ frequencies did not decline with age (r = -0.05, p = 0.83; n = 34; Fig 1E). Together these data suggest that exposure to malaria parasites may contribute to a loss of peripheral blood T_regs_ in this high transmission setting.

### Longitudinal decline in T_reg_ frequencies within children correlates with higher intercurrent malaria exposure

**Fig 2:**
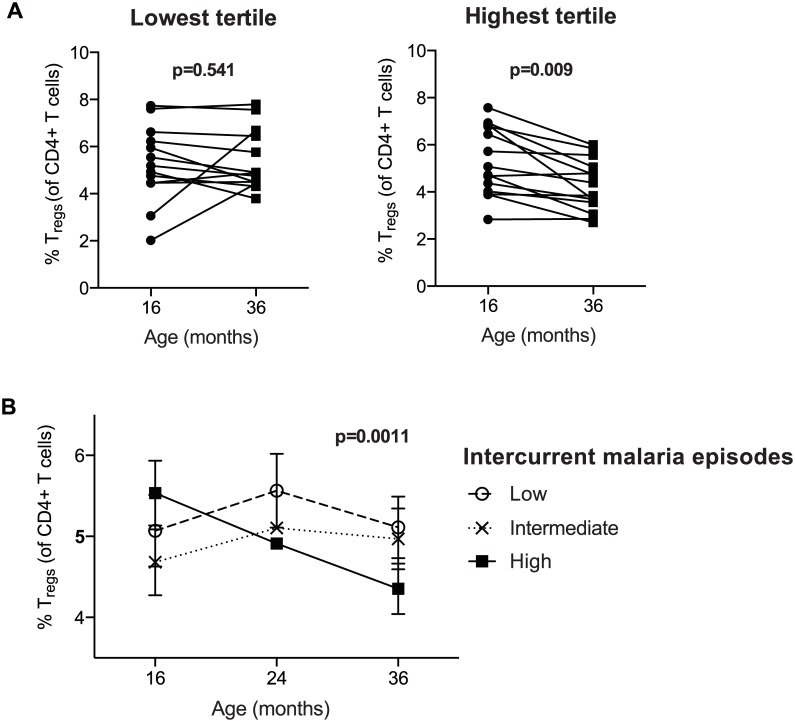
Regulatory T cells decrease over time in individuals with high but not low malaria incidence. T_reg_ frequencies of CD4+ T cells (FoxP3+CD25+CD127^dim^) were measured at 16, 24 and 36 months of age (PROMOTE- SP arm). **(A)** Children were divided into tertiles based on incidence of malaria between 16 and 36 months; lowest tertile median incidence 4.0 episodes ppy (IQR 3.0–5.5); intermediate tertile median incidence 8.2 episodes ppy (IQR 7.3–9.2); highest tertile median incidence 13.7 episodes ppy (IQR 10.3–14.6). The median duration since last malaria infection in these three tertiles was 8.5, 20, and 100 days, respectively. Wilcoxon matched pairs signed rank test p values indicated. Between 16 and 36 months, T_reg_ frequencies declined in individuals in the highest but not lowest tertile of malaria incidence. **(B)** Changes in T_reg_ frequencies between 16, 24 and 36 months were compared between children in the lowest, intermediate, and highest tertiles of malaria exposure by generalized estimate equations, accounting for repeated measures, age, duration since last malaria episode and parasite status at time of sampling.

To investigate whether changes in T_reg_ frequencies within individual subjects over time correlates with their malaria incidence, we measured T_reg_ frequencies longitudinally in 41 subjects at 16, 24 and 36 months of age (PROMOTE cohort, SP chemoprevention arm). Subjects were stratified into tertiles based on their number of malaria infections between 16 and 36 months. Among children in the highest tertile of malaria incidence (n = 14), there was a consistent decline in T_reg_ frequencies between 16 and 36 months of age (Wilcoxon signed rank test, p = 0.009, Fig 2A). In contrast, among children in the lowest tertile of incidence (n = 13), there was no change in T_reg_ frequencies between 16 and 36 months (Wilcoxon signed rank test p = 0.54). Repeated-measures analysis using generalized estimating equations confirmed that changes in T_reg_ frequencies over time differed between the exposure groups (p = 0.0011, Fig 2B). Together, these data suggest that very high malaria exposure during childhood results in the loss of peripherally circulating T_regs_ within individuals over time.

### T_reg_ dynamics during childhood differ between high and low malaria transmission settings

**Fig 3:**
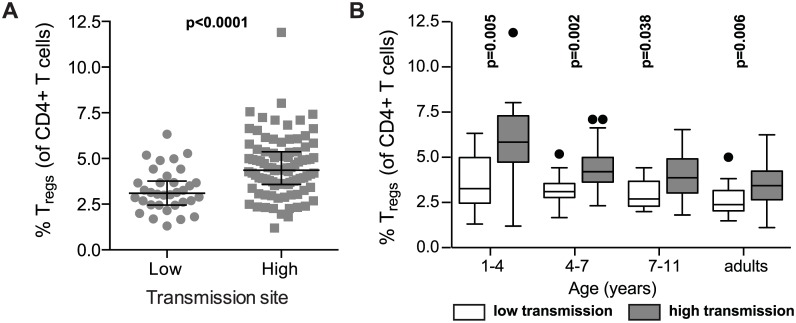
FoxP3+ regulatory T cells are increased in high compared to low transmission settings, but decrease with age only in highly exposed children. **(A)** FoxP3+CD25+CD127^dim^ regulatory T cell frequencies in children age 1 to 11 years (PRISM cohort) residing in the low transmission Jinja District (n = 34) were compared to children in the high transmission Tororo District (n = 91). Wilcoxon ranksum p value indicated. **(B**) FoxP3+CD25+CD127^dim^ regulatory T cell frequencies were compared between children from low and high transmission areas at age 1–4 (n = 11 and n = 18), 4–7 (n = 14 and n = 35) and 7–11 (n = 9 and n = 38) years of age and adults (n = 9 and n = 37). Wilcoxon ranksum for age group comparisons, p values indicated.

Prior studies have shown that experimental and natural *P*. *falciparum* infection induces the expansion of regulatory T cells *in vivo* [4,5]. To investigate whether and how T_reg_ dynamics differ between settings of high and low exposure, we compared T_reg_ frequencies between children over a range of ages in the high transmission district of Tororo to children from the low transmission district of Jinja (PRISM cohort age 1 to 11 years). Children in the high transmission Tororo District experienced a much higher malaria incidence (median 3.6 vs. 0 ppy) and a much shorter duration since last infection (median 62 vs. 230 days) than children in the low transmission Jinja District (full details in S1 Table). Overall, T_reg_ frequencies (FoxP3+CD25+CD127^dim^) were higher in children from the high transmission district compared to the lower transmission district across all age groups (Wilcoxon ranksum p<0.0001, Fig 3A), and this difference was most marked in the youngest age group (Fig 3B), possibly reflecting an earlier expansion of T_regs_ in response to initial infections during early childhood or even *in utero* [27–30]. The difference in T_regs_ frequencies between the low and high transmission study sites decreased with increasing age, and this trend extended to adulthood (Fig 3B). Thus, our data suggest that in areas of high malaria transmission malaria infections early in life induce T_regs,_ as has been previously described among naïve or comparatively low-exposure individuals [4,5,7,8]. However, in areas of intense and continual malaria exposure, parasite-driven induction of T_regs_ is diminished, and instead there is a progressive decline of T_regs_ with repeated malaria episodes. This decline does not appear to be transient, as T_reg_ frequencies do not correlate with the duration of time since last malaria episode or asymptomatic parasite infection. Instead, there appears to be sustained and progressive loss of T_regs_ with age among children heavily exposed to malaria.

### Reduced TNFR2 expression on T_regs_ following chronic malaria exposure

**Fig 4:**
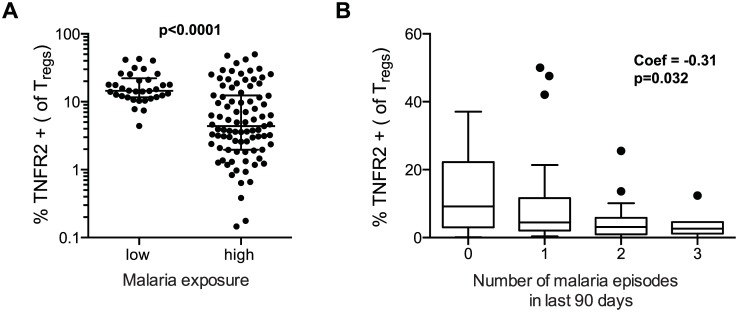
TNFR2 expression on FoxP3+ regulatory T cells declines with increasing prior malaria incidence. **A.** Frequencies of TNFR2 expressing T_regs_ (FoxP3+CD25+CD127^dim^) were compared between children in the high malaria transmission area (Tororo District) and in the low transmission area (Jinja district). TNFR2 expression on T_regs_ was higher in low compared to high transmission settings. Wilcoxon signed rank test indicated. **B.** The association between frequencies of TNFR2 expressing T_regs_ and number of recent malaria episodes was analyzed among children from high malaria exposure area (Tororo District). TNFR2 expression declined with increasing number of malaria episodes in last 90 days. Regression coefficient and p value are indicated.

We next assessed expression of TNFR2 on T_reg_ cells, as this receptor has been shown to be critical for both proliferative expansion of T_regs_ and maintenance of *FOXP3* expression in inflammatory environments [31,32]. Furthermore, T_regs_ expressing TNFR2 have been shown to have enhanced suppressive capacity [8,33,34], and are increased during malaria infection [8]. We found that the percentage of T_regs_ expressing TNFR2 was significantly lower among PRISM cohort children from the high transmission Tororo District than among children of similar age from the low transmission Jinja district (p<0.0001, Fig 4A, see S3 Fig for gating strategy). Among Tororo children, expression of TNFR2 was inversely correlated with number of recent malaria episodes (Coef = -0.31, p = 0.032, Fig 4B), although expression was slightly higher on T_regs_ from children currently PCR-positive for *P*. *falciparum* infection (p = 0.043). These data suggest that TNFR2 expression is transiently up-regulated during parasitemia but declines over time following repeated malaria episodes. This decrease in TNFR2 expression could contribute to the loss of FoxP3+ T_regs_ from peripheral blood by decreasing the stability of *FOXP3* expression [31,32].

### Altered homeostasis of T_regs_ following intense malaria exposure

**Fig 5:**
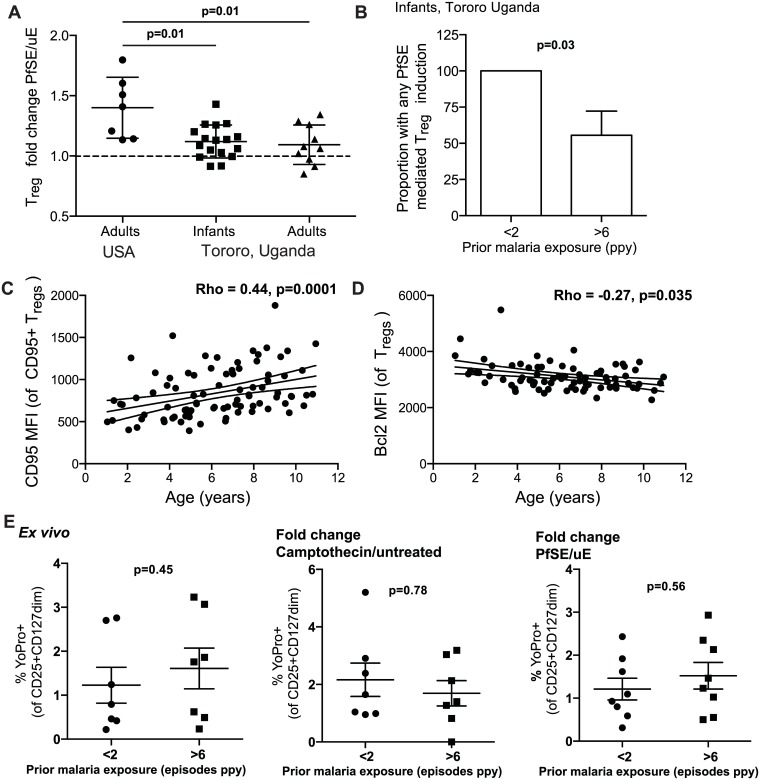
Evidence for changed homeostasis of T_regs_ in children with high malaria exposure. **A.** PBMCs from 28 month old children (PROMOTE no chemoprevention control arm), malaria-exposed adults (PRISM cohort, Nagongera, Tororo District) and naïve adults were incubated for 7 days with protein extract from mature stage *P*. *falciparum* infected RBCs (PfSE) or uninfected RBCs (uE). T_reg_ frequencies were enumerated (FoxP3+CD25+CD127^dim^) and induction factor was calculated based on frequency fold change between PfSE and uE stimulated PBMCs. Parasite induction of T_regs_ was reduced in exposed compared to naïve individuals. Wilcoxon signed rank test indicated for comparison between naïve-adults and exposed infant and adult samples. **B.** The proportion of children with any T_reg_ induction by PfSE was compared between those with low (<2 episodes ppy, n = 10) and high (>6 episodes ppy, n = 10) prior malaria incidence. There was a reduced proportion of infants with any T_reg_ induction in those who had high compared to low prior malaria incidence. Chi-square test indicated. **C.** Pro-apoptotic marker CD95 was measured on T_regs_ in children (PRISM cohort) from the high transmission area (Tororo District). Association between the level of CD95 expression on T_regs_, as measured by MFI of CD95+ T_regs_, and age was analyzed. The level of CD95 expression increased with age. **D.** Pro-survival Bcl2 was measured on T_regs_ in children (PRISM cohort, Nagongera, Tororo) from the high transmission area. Association between the expression of Bcl2 on the T_reg_ population and age was analyzed. The level of Bcl2 expression decreased with age. Spearman’s rho and p indicated. **E.** YoPro staining of T_regs_ from 28 month old children (PROMOTE, no chemoprevention control arm, with low (<2 episodes ppy) and high (>6 episodes ppy) prior malaria incidence was measured ex vivo and following stimulation with camptothecin (an activator of apoptosis) or *P*. *falciparum* antigen. Data from YoPro staining is representative of all measures of apoptosis (see S5 Fig additional measures of apoptosis including activated Caspase 3 and AnnexinV). There was no difference in sensitivity to apoptosis between infants with low and high prior malaria incidence. Wilcoxon signed rank test indicated.

The progressive decline in circulating T_regs_ in children heavily exposed to malaria could be explained by changes in T_reg_ homeostasis such as decreased induction, increased loss (due to apoptosis or downregulation of *FOXP3*), or both. Therefore, we next examined whether heavy prior malaria infection altered the propensity for T_reg_ induction or apoptosis. It has previously been shown that *in vitro* stimulation of adult PBMCs with *P*. *falciparum* antigen induces regulatory T cells [9,10,12,35]. To investigate whether the propensity of CD4 T cells to differentiate into T_regs_ in response to parasite antigen is influenced by age and/or prior malaria exposure, we measured induction of T_regs_ following *in vitro* stimulation with *P*. *falciparum* schizont extracts (PfSE) in malaria-naïve adults, malaria-exposed children (28 months of age), and malaria-exposed adults from the high incidence district of Tororo (gating strategy and *ex vivo* T_reg_ frequencies shown in S4 Fig). As previously reported, co-culture of PBMCs from naïve adults with PfSE resulted in consistent induction of T_regs_ (Fig 5A and S4C Fig). However, using PBMC from malaria-exposed children and adults, induction of T_regs_ was reduced compared to naïve adults (Fig 5A). Further, whereas all children with low prior malaria exposure (<2 episodes ppy) exhibited T_reg_ induction (fold change >1), only 55% of children with high prior malaria exposure (>6 episodes ppy) induced T_regs_ following PfSE stimulation (p = 0.03; Fig 5B). This suggests that heavy prior exposure to malaria may limit the propensity of CD4 cells to differentiate into T_regs_ upon re-encounter with parasite antigens.

We next investigated whether heavy prior malaria exposure increased the susceptibility of T_regs_ to apoptosis, as has been shown in chronic HIV-1 infection [36]. The percentage of T_regs_ expressing the pro-apoptotic marker CD95 increased with age among Tororo children (Rho = 0.175, p = 0.079), as did the level of CD95 expression (as calculated by MFI of CD95 on CD95+ T_regs_, Spearman’s Rho = 0.44, p = 0.0001) (Fig 5C). Conversely, expression of the anti-apoptotic marker Bcl2 on T_regs_ declined with age (Rho = -0.266 p = 0.035) (Fig 5D). However there was no independent relationship between expression of these markers and prior malaria incidence, current parasite infection, nor time since last malaria episode, suggesting that age may independently affect the sensitivity of T_regs_ to apoptosis. To further investigate this, three distinct measures of apoptosis (YoPro, Annexin V and activated Caspase 3) were measured on T_regs_ both *ex vivo* and following stimulation with camptothecin (an activator of apoptosis) or *Pf*SE in 28-month infants with low or high prior malaria incidence (PROMOTE no-chemoprevention control arm). There was no difference in sensitivity to apoptosis as measured by any of the markers either *ex vivo* or following stimulation with camptothecin or parasite antigen; the frequencies of positively stained cells *ex vivo*, and the fold change of apoptosis staining, were the same regardless of prior malaria exposure (Fig 5E and S5 Fig). Together these data suggest that T_reg_ homeostasis may be altered in the setting of heavy malaria exposure, in part due to reduced induction of peripheral T_reg_ cells, with little evidence for increased susceptibility to antigen-driven apoptosis.

### Lower T_reg_ frequencies may be associated with a decreased risk of symptoms following parasite infection

We finally asked whether the frequency of circulating T_regs_ influences susceptibility to malaria. We assessed the influence of T_reg_ frequencies on protection from malaria in both the 2-year-old PROMOTE no chemoprevention control arm and 4-year-old TCC cohorts using two methods; a time-to-event analysis (time to next malaria episode), and negative binomial regression of the relationship of T_reg_ frequencies to malaria incidence in the year following assessment. Among 2-year-olds, we found that higher T_reg_ frequencies were associated with an increased time to next malaria episode and a lower future malaria incidence in univariate analysis (Table 1). However, after adjusting for prior malaria incidence in a multivariate model to account for heterogeneity in environmental exposure to infected mosquitoes [22,37,38], this relationship was no longer significant, suggesting that differences in environmental exposure intensity may underlie this association [37]. Among 4-year-olds, T_reg_ frequencies were not associated with time to next malaria infection or malaria incidence during follow-up. Similarly, no relationships between T_reg_ frequencies and malaria incidence in follow-up or time to next malaria episode were observed in the PRISM 1–11 year old cohorts, in either the low or the high transmission study sites, even after adjustment for household mosquito exposure. Thus we did not find clear evidence that T_regs_ are associated with the risk of clinical malaria.

**Table 1:** Relationship between T_reg_ frequencies and prospective risk of malaria.

	Future malaria incidence				Time to next malaria episode			
	Univariate		Multivariate^1^		Univariate		Multivariate^1^	
	IRR	p	IRR	p	HR	p	HR	p
2yo (n = 81)	0.92	**0.006**	0.96	0.11	0.83	**0.003**	0.9	0.09
4yo (n = 75)	0.95	0.3	1.02	0.5	0.92	0.236	1.05	0.526

^1^ –Multivariate is adjusted for prior malaria incidence

Given their immunoregulatory role, is also possible that T_regs_ play a role in protecting the host from the symptomatic manifestations of malaria once *P*. *falciparum* infection is established [1,2,15,19]. To assess whether T_regs_ may influence the risk of clinical disease once infected, we analyzed the relationship between T_reg_ frequencies and the probability of symptoms once parasitemic using generalized estimate equations with robust standard errors, accounting for repeated measures [16,39]. Comparing children with the lowest compared to highest tertiles of T_regs_, lower T_reg_ frequencies were associated with an increased monthly probability of infection, consistent with the exposure induced decline in T_regs_ described. However, lower T_reg_ frequencies were also associated with an overall decreased probability of becoming symptomatic once infected (2 year old PROMOTE cohort; OR = 0.4, p = 0.039, 4 year old TCC cohort; OR = OR = 0.37, p = 0.06), suggesting that the decline in circulating Tregs may be associated with the acquisition of clinical immunity.

## Discussion

Here, we have shown through both cross-sectional and longitudinal studies that the percentage and absolute number of FoxP3+ T_regs_ in peripheral blood are influenced by repeated exposure to malaria. While children in settings of intense exposure have higher T_reg_ frequencies during early childhood, frequencies decline throughout childhood in settings of high (but not low) exposure, and the extent of T_reg_ loss correlates with the intensity of *P*. *falciparum* exposure. We provide both *in vivo* and *in vitro* evidence that among children in high exposure settings, there is a reduction of parasite induced T_reg_ expansion during infection. Further, we show a down-regulation of TNFR2, which is required for stabilization of the FoxP3+ regulatory phenotype in inflammatory environments [31]. These data demonstrate that chronic exposure to malaria results in altered T_reg_ homeostasis *in vivo*, which may have a downstream impact on the acquisition of immunity and control of infection.

Our data indicate that the dynamics of T_reg_ induction and homeostasis differ markedly between high and low malaria transmission settings. Although children residing in high transmission areas had higher T_reg_ frequencies overall, perhaps in response to a parasite-driven T_reg_ expansion in early childhood or *in utero* [27–30], we observed a marked *decline* in T_reg_ frequencies beginning at 1–2 years of age, which appeared to be driven by persistent parasite exposure. Further, among highly exposed children, we saw no association between T_reg_ frequencies and current or recent infection, suggesting that T_regs_ have reduced *in vivo* induction during infection of these chronically exposed children. Consistent with this, we demonstrated that induction of T_regs_ following parasite stimulation of PBMC was diminished in heavily exposed adults and children, providing *in vitro* evidence that chronic antigen exposure may blunt the proliferative expansion of T_regs_ in response to malaria. This is in contrast to published studies suggesting that T_regs_ expand in response to malaria *in vivo* and *in vitro* [4,7–13,18,19]. The most likely explanation for the difference in our findings is that these earlier studies were performed largely on malaria-naïve volunteers or relatively low-exposed populations. Overall our data suggest that while T_regs_ may be induced in initial encounters with parasites, induction capacity is diminished after repeated parasite exposure and instead T_regs_ undergo a steady decline in the periphery. The induction of T_regs_ by *Plasmodium* is believe to occur through activation of latent membrane-bound TGFβ [11,40], which can be blocked by antibodies to the *P*. *falciparum* thrombospondin-related adhesive protein (*Pf*TRAP) [11]. This T_reg_ induction mechanism is shared by related protozoal pathogens *Toxoplasma* and *Leishmania* [35] and may represent an immune evasion strategy. The reduced capacity of parasite antigen to induce T_regs_ in heavily malaria exposed children suggests that the host may be able to circumvent parasite induction of T_regs_, potentially enabling enhanced control of infection.

Another potential mechanism for the observed decline in T_regs_ among children chronically exposed to malaria is via loss of *FOXP3* expression by “unstable” T_regs,_ which has been reported to occur in highly inflammatory immune environments [41–45]. Sustained expression of the canonical transcription factor *FOXP3* by T_regs_ is critical for maintenance of regulatory function [46]. Several recent studies suggest that T_regs_ can become “unstable” and lose *FOXP3* expression in response to cues in the microenvironment, although the significance, extent, and triggers of this phenomenon remain subject to considerable debate [44,45,47–52]. Lineage tracking experiments have elegantly shown that antigen-driven activation and inflammation can drive a subset of FoxP3^hi^ T_regs_ to lose both *FOXP3* expression and suppressor function [44], and even acquire an effector phenotype [53]. Further, repeated TCR stimulation leads to the loss of *FOXP3* expression and the conversion to pro-inflammatory cytokine producing cells in natural T_regs_ *in vitro* [54]. Our data suggest a potential mechanism for such T_reg_ destabilization in malaria infection, as recurrent exposure resulted in down-regulated T_reg_ expression of TNFR2, which has been shown to be critical for both the proliferative expansion of T_regs_ and stabilization of their *FOXP3* expression in inflammatory environments [31,32,55]. In the setting of malaria, TNFR2+ T_regs_ have previously been shown to have higher *FOXP3* expression and enhanced suppressive function [8]. Thus our data are consistent with mounting evidence suggesting that peripherally induced T_regs_ have significant plasticity in response to inflammatory environments such as that observed in malaria infection, which may culminate in loss of *FOXP3* expression and suppressive function.

Several additional processes might contribute to the observed loss of T_regs_ in peripheral blood. Because T_regs_ track to sites of inflammation, it is possible that T_regs_ induced by *P*. *falciparum* traffic to the liver, spleen, or secondary lymphoid organs during infection. Invasive sampling was not possible in our study cohorts; therefore we were unable to exclude a preferential sequestration of T_regs_ in tissues or lymphoid organs. However, we did not observe any statistical relationship of T_reg_ frequencies with the presence of parasitemia, nor with the amount of time elapsed since the last *P*. *falciparum* infection, as might be expected if T_regs_ migrate to sites of local inflammation during active infection. Alternatively, loss of T_regs_ through apoptosis might contribute to their decline following repeated malaria infections. However, we observed no relationship between prior malaria incidence and the expression of the pro-apoptotic molecule CD95 or the anti-apoptotic marker Bcl2 on T_regs_ (after controlling for age), nor did we observe a differential susceptibility towards apoptosis *ex vivo*, or following *in vitro* re-stimulation with parasite antigens.

The observed decline in T_reg_ frequencies with increasing prior malaria incidence contrasts with that of another regulatory T lymphocyte population, IL10-producing Th1 cells, which we have recently shown to dominate the *P*. *falciparum*-specific CD4 T cell response among heavily malaria-exposed children, including among children from both the TCC and the PRISM Nagongera, Tororo cohorts tested here [22,56]. This “autoregulatory” population consists predominantly of IL10/IFNγ co-producing cells that express the canonical Th1 transcription factor Tbet and appear to be short-lived in the periphery, exhibiting a strong association with recent infection [22]. We observed no statistical relationship between frequencies of IL10-producing Th1 cells and conventional FoxP3+ T_regs_, in contrast to an earlier small cohort study that reported a positive correlation between these two regulatory cell populations [57]. This suggests that in highly exposed children, the loss of peripherally circulating T_regs_ is not directly compensated by increased frequencies of IL10 producing CD4 responses. In addition, we did not observe any statistical relationships between T_reg_ frequencies and *P*. *falciparum*-specific CD4 effector responses. In prior studies, *FOXP3* mRNA levels measured during acute malaria were shown to correlate inversely with the magnitude of the subsequent Th1 memory response to *P*. *falciparum* measured 28 days after infection [6]. Similarly, *FOXP3* expression among malaria-naive adults following experimental sporozoite vaccination was shown to correlate inversely with the Th1 memory response measured >100 days later [14]. Thus, while T_regs_ are likely to influence the development of parasite-specific T cell memory responses, no relationship between these populations could be demonstrated through our concurrent measurements in peripheral blood, which maybe due in part to the chronicity of malaria exposure in these children and/or the substantial heterogeneity within the cohort with regard to time elapsed since the last infection. Furthermore, additional parameters of T_reg_ function that cannot readily be measured in peripheral blood in large cohorts, such as suppression of T cell proliferation and modulation of APC function, are likely to influence the cellular immune response to malaria, but could not be assessed in the present study.

While our results clearly suggest that repeated malaria impacts peripherally circulating T_regs_ in children, the role of these cells in protection from malaria and the development of immunity remains unclear. We observed no association between T_reg_ frequencies and future malaria incidence or time to next malaria episodes in any of our cohorts. However, our data suggest that although children with the lowest T_reg_ frequencies had a higher monthly probability of infection, they were less likely to become symptomatic once infected compared to children with the highest T_reg_ frequencies over the entire study period. While these data suggest that clinical immunity is acquired as T_regs_ decline, the role of T_regs_ in mediating clinical immunity remains unclear, and may not be causal—rather, declining T_reg_ frequencies may coincide with other immune changes that mediate protection. Because all children in our study cohorts have easy access to dedicated study clinics and prompt antimalarial drug treatment, the incidence of severe malaria was extremely low, preventing assessment of the potential role of T_regs_ in protection from severe disease. We were similarly unable to assess the impact of T_reg_ activity on pathogen persistence following infection, because all cases of symptomatic malaria were promptly treated with potent artemisinin-based drugs, thus altering the natural course of infection. In other protozoal infections, such as leishmaniasis and toxoplasmosis, pathogen-induced T_regs_ have been reported to curb the inflammatory response, allowing long-term pathogen persistence [1,2,15]. Indeed, in murine models of leishmania, pathogen persistence resulting from T_reg_-mediated immune suppression has been shown to be a requirement for immunity to re-infection [1]. The long-term asymptomatic maintenance of low-burden *P*. *falciparum* infection that is commonly observed among adults in high-transmission areas [58] may represent a similar phenomenon, but the role of T_regs_ in mediating this process is not known. Although we did not observe higher frequencies of peripheral blood T_regs_ among children with asymptomatic *P*. *falciparum* infection, which is not routinely treated in Uganda, this does not exclude a role for T_regs_ in maintaining this state of host-parasite equilibrium.

In conclusion, we observed a progressive loss of T_regs_ from the peripheral blood of children following chronic repeated malaria infections, accompanied by downregulation of TNFR2 and diminished *in vitro* induction of T_regs_ by parasite antigen. Together these data demonstrate that the impact of chronic malaria antigen exposure on the FoxP3+ regulatory T cell population is quite different from that of acute infection of malaria-naïve individuals. Our findings also add to mounting data suggesting that the stability and homeostasis of FoxP3+ T_regs_ are perturbed under highly inflammatory conditions. The implications of this pathogen-driven T_reg_ loss for pathogen clearance, host-parasite equilibrium, and the development of clinical immunity in regions of intense malaria transmission require further investigation.

## Materials and Methods

### Ethics approval

Written informed consent was obtained from the adult individual or parent/guardian of all study participants. Study protocols were approved by the Uganda National Council of Science and Technology and the institutional review boards of the University of California, San Francisco, Makerere University and the Centers of Disease Control and Prevention.

### Study participants

Samples for this study were obtained from children enrolled in 3 longitudinal childhood malaria cohort studies conducted in Tororo District and Jinja District of eastern Uganda. Cohort characteristics are described in S1 Table. For all cohorts, samples were selected on the bases of availability of PBMCs.

1. The PROMOTE-Chemoprevention Study was conducted from 2010–2013 and enrolled 400 children who were randomized to receive chemoprevention with monthly sulfadoxine-pyrimethamine (SP), daily trimethoprim-sulfamethoxazole (TS), monthly dihydroartemisinin-piperaquine (DP) or no chemoprevention (control arm) from 6–24 months of age, then followed for an additional year after the intervention ended. Results of this trial have been published [59]. In this report we only include data from children who were randomized to receive “no chemoprevention (control arm)” or SP, which was found to have no efficacy for prevention of malaria [59]. Samples from this cohort were taken at 16, 24, 28 and 36 months of age as indicated.
2. The Tororo Child Cohort Study (TCC) was conducted from 2007–2012 in Tororo district, and enrolled children at approximately 6 months of age and followed until age 5. Results of this study have previously been published [60]. Samples used here are from patients who were HIV-negative children born to HIV-negative mothers, taken at age 4 years.
3. The PRISM cohort was initiated in 2011 and is ongoing. This longitudinal observational cohort consists of 200 households across two study sites, the Nagongera sub-county in Tororo district and the Walukuba sub-county in Jinja district. Description of the study and results have been published in [21] In all households, one adult caregiver and all eligible children aged 6 months to 11 years were enrolled into the study. Samples used here were taken from cross-sectional bleeds of study participants taken between August 2013 and March 2014. Household-level mosquito exposure was calculated based on mosquito counts obtained from CDC light traps placed monthly within the household of each individual trial participant in the 2012 [26].

For the PROMOTE-Chemoprevention, TCC and PRISM Nagongera high transmission area, the estimated entomological inoculation rate (aEIR) is approximately 310 bites ppy. In contrast, at the PRISM Walukuba low transmissions site the aEIR is estimated at 2.8 [21].

### Clinical management and measurement of malaria incidence

On enrollment all study participants were given an insecticide treated bed net and followed for all medical care at dedicated study clinics. Children who presented with a fever (tympanic temperature ≥38.0°C) or history of fever in the previous 24 hours had blood obtained by finger prick for a thick smear. If the thick smear was positive for malaria parasites, the patient was diagnosed with malaria regardless of parasite density and treatment with artemether-lumefantrine or dihydroartemisinin-piperaquine for all episodes of malaria. Incident episodes of malaria were defined as all febrile episodes accompanied by any parasitemia requiring treatment, but not preceded by another treatment in the prior 14 days. The incidence of malaria was calculated as the number of episodes per person years (ppy) from the time of enrolment into the cohort. In a subset of PRISM cohort children used to assess TNFR2 expression on T_regs_ parasite infection was assessed via PCR from dried blood spots as previously described [61].

### FoxP3+ Regulatory T cells measurements and *P. falciparum* specific CD4 responses

T_reg_ frequencies were enumerated from whole blood and fresh and cryopreserved PBMCs as indicated below. For enumeration of T_regs_ from whole blood (PROMOTE-Chemoprevention, control arm 2-year-old samples), 100 μl of fresh whole blood was stained with BD Pharmingen anti-CD3-FITC (UCHT1), anti-CD4-PE-CY7 (SK3), and CD25-APC (M-A251) for 20 minutes and then lysed and permeabilized with eBioscience RBC lysis buffer. Cells were washed and then incubated with eBioscience anti-FoxP3-PE (PCH101). Samples were acquired on Accuri C6 Cytometer.

For analysis of T_regs_ from fresh PBMCs (PRISM Nagongera cohort), PBMCs were isolated by Ficoll density gradient centrifugation and rested over night in 10% fetal bovine serum. PBMCs were stained with BD Pharmingen anti-CD3 PerCP (SK7), anti-TNFR2-Alexa646 (hTNFR-M1), anti-CD95-PECy7 (DX2) and Biolegend anti-CD4-APC-Cy7 (OKT4), anti-CD25-BrillantViolet510 (M-A251), anti-CD127-PacificBlue (A019D5). Following surface staining, cells were fixed and permeabilized with eBioscience FoxP3 staining set and intracellular stained with FoxP3-PE (PCH101) and BD Pharmingen anti-Bcl2-FITC (Bcl-2/100) as per manufacturers protocol. Samples were acquired on three laser BD FACsCantoII with FACSDiva software.

For analysis of T_regs_ from frozen PBMCs (Tororo Child Cohort 4-year-olds, PROMOTE-Chemoprevention SP arm longitudinal samples at 16, 24, and 28 months of age, PRISM Walukuba cohort), cryopreserved PBMCs were thawed using standard methods, and immediately stained with the following panels of antibodies; BD Pharmingen anti-CD3-FITC (UCHT1), anti-CD4-PE-CY7 (SK3), CD25-APC (M-A251) and Biolegend anti-CD127 Pacific Blue (A019D5); or Biolegend anti-CD3-BrilliantViolet650 (OKT3), anti-CD4-PerCP (OKT4), anti-CD127-FITC (A019D5); or Biolegend anti-CD3-PerCP (OKT3), anti-CD4-APC-Cy7 (OKT4), anti-CD25-BrillantViolet510 (M-A251), anti-CD127-PacificBlue (A019D5), anti-TNFR2-APC (3G7A02). Live/dead aqua amine (Invitrogen) was included in all panels. Following surface staining, cells were fixed and permeabilised with eBioscience FoxP3 staining set and intracellular stained with FoxP3-PE (PCH101) as per manufacturers protocol. Samples were acquired on LSR2 three laser flow cytometer (Becton Dickinson) with FACSDiva software.

For calculation of absolute T_regs_ counts (i.e. cells per μl, PRISM Nagongera cohort), peripheral blood CD4 T cell concentrations were measured from whole blood using counting beads, and T_reg_ frequencies were calculated by normalization to total CD4 T cell numbers.

### CD4 T cell responses to *P. falciparum* infected RBCs

Analysis of CD4+ T cell responses to *P*. *falciparum* infected RBCs via intracellular cytokine staining was performed as previously described [22,56]. PBMCs were stimulated with *P*. *falciparum* infected RBCs or uninfected RBCs and CD4 T cell production of IFNγ, IL10, and TNFα were measured via intracellular staining.

### Induction of Regulatory T cells by *P. falciparum* in vitro

PBMCs from PROMOTE subjects (28 months of age; no chemoprevention control arm) and adults from the high malaria transmission region of Tororo were thawed and washed in 10% Human serum (AB) media (Gemini), and 3–6X10^6^ PBMC were labeled with 1 ml of 1.25 mM 5,6-carboxyfluorescein diacetate succinimidyl ester (CFSE; Molecular Probes) for seven minutes. CFSE-labeled PBMC were incubated in 96-well, deep-well culture plates (Nunc, Roskilde, Denmark) at 10^6^ PBMC/ well in 1 ml for 7 days with *P*. *falciparum* schizont extract (PfSE) (W2 strain) or protein extract from uninfected RBCs (uE) at a effector to target ratio equivalent to 1:1 PBMC:infected RBC. PHA (1μg/ml) was used as a positive control. PfSE extracts were made from the W2 stain grown in standard culture conditions and confirmed to be free of mycoplasma contamination using MycoAlert (Lonza). Mature stage parasites were magnet purified from culture using MACs purification columns. Purified parasites or uninfected RBCs were freeze thawed 3+ times (via snap freezing on liquid nitrogen and then transfer to 37°C water bath) to produce PfSE and uE and stored at -20°C. Following culture of PBMCs with protein extracts, cells were treated with 100 units of DNase I (Invitrogen) in culture media for 5 minutes and then surface stained with Biolegend anti-CD3-BrilliantViolet650 (OKT3), anti-CD4-PerCP (OKT4), anti-CD25-PE-Cy7 (BC96), anti-CD127 Pacific Blue (A019D5), BD Pharmingen anti-CD8-ABC-H7 (SK1) and Live/dead aqua amine (Invitrogen cells). Following surface staining, cells were fixed and permeabilized with eBioscience FoxP3 staining set and stained with FoxP3-PE (PCH101). Proliferation with PHA was used to ensure cell viability, and cells incubated with uE were used as a background control. Of the infants tested, the prior median malaria incidence was 1.2 episodes ppy in the low exposed group and 8.5 episodes ppy in the high exposed group.

### Susceptibility of T_regs_ to apoptosis

PBMCs from PROMOTE subjects (28 months of age; no chemoprevention control arm) were thawed and rested overnight either in standard media (untreated), 5uM camptothecin (Sigma) or *P*. *falciparum* schizont extract (PfSE) or protein extract from uninfected RBCs (uE) at an effector:target ratio of 3:1. To test for induction of apoptosis, stains for AnnexinV (Biolegend) or YoPro (Invitrogen), or activated Caspase 3 FITC (BD) were used according to the manufacturer’s instructions in combination with the following antibodies: AnnexinV and YoPro staining—CD3 (OKT3) Brilliant Violet 650, CD4 (RPA-T4) APC-Cy7, CD127 (A019D5) APC, CD25 (BC96) PE-Cy7 from Biolegend; for Caspase3—CD3 (OKT3) Brilliant Violet 650, CD4 (RPA-T4) PerCP, CD127 (A019D5) Pacific Blue, CD25 (BC96) PE-Cy7. T_regs_ were gated as CD3+CD4+CD25+CD127^dim^. Sensitivity to apoptosis was measured *ex vivo* (untreated control), after induction with camptothecin (fold change compared to untreated), and after stimulation with *P*. *falciparum* schizont extract (fold change comparing PfSE to uE).

### Flow cytometry data analysis

Unless otherwise indicated, samples were acquired on an LSR2 flow cytometer (Becton Dickinson) with FACSDiva software. Flow cytometry data were analysed using FlowJo software (Tree Star, San Carlos, CA). Color compensation was performed using single color cell controls or beads stained for each fluorochrome. Gating strategies are outlined in Supplementary Figures. Fluorescence minus one controls were used for gating of CD25, CD95, HLA-DR and Bcl2. For FoxP3 staining, an anti-Rabbit-Isotype control was used.

### Statistical analysis

Data analysis was performed using Stata version 12 (Stata Corp, College Station, Tx) and PRISM version 6 (Graph Pad). Associations between T_reg_ frequencies and other continuous variables (prior malaria incidence, age, time since last malaria episode) were assessed using Spearman’s correlation. Changes in T_reg_ frequencies within an individual over time were assessed using the Wilcoxon signed rank test. All other two-group comparisons of continuous variables were performed using the Wilcoxon rank sum test. Repeated measures analysis of longitudinal changes in T_reg_ frequencies was performed using generalized estimating equations, with adjustment for concurrent parasitemia, age and duration since last malaria episode. Categorical variables were compared using Chi sq test. Associations between T_reg_ frequencies and time to next malaria episode were evaluated using the Kaplan-Meier product limit formula, and a multivariate cox proportional hazards model was used to adjust for surrogates of malaria exposure (cumulative episodes since enrollment in study for TCC and PROMOTE cohorts, or age for PRISM cohorts). Negative binomial regression was used to estimate associations between T_reg_ frequencies and the prospective incidence of malaria in the following year (incidence rate ratios, IRR) and prevalence of asymptomatic parasitemia in the following year (prevalence rate ratios, PRR), adjusting for malaria exposure as above. Two-sided p-values were calculated for all test statistics and p<0.05 was considered significant. In the PRMOTE and TCC cohorts, associations between the highest and lowest tertiles of T_reg_ frequencies and the monthly risk of parasitemia, probability of symptoms if parasitemic, and incidence of malaria, stratified by year of age, were evaluated using generalized estimating equations with robust SEs accounting for repeated measures in the same patient, for the period of the inter study (6 months to 3 or 5 years of age) [62].

## Supporting Information

S1 Table Cohort characteristics. (PDF)

S1 Fig Gating strategy for regulatory T cells. Frequencies of regulatory T cells were enumerated by staining whole blood samples (2yo cohort) or frozen PBMC samples (4yo cohort). **A.** For 2yo, whole blood was stained and cells analyzed on four-color Accuri flow-cytometer. **B.** For 4yo samples, PBMCs were thawed and stained and analyzed on a LSRII. See also Fig 1A and 1B.
(PDF)

S2 Fig Absolute count of regulatory T cells decline with increasing age among children in a high transmission setting. Absolute count of regulatory T cells, analyzed as the percent of FoxP3+CD25+CD127^dim^ expressing CD4+ T cells, normalized to CD4+ T cell absolute counts, from 1 to 11 year old children (PRISM cohort, high transmission Nagongera, Tororo District), declined with increasing age.
(PDF)

S3 Fig Gating strategy for TNFRII expression. Frequencies of regulatory T cells expression TNFR2 were enumerated by staining PBMCs. Gating for FoxP3+CD25+CD127dim regulatory T cells was as for S1 Fig. TNFR2 staining was gated on FMO controls, as indicated.
(PDF)

S4 Fig Regulatory T cell induction assays. **A.** PBMCs were incubated with *P*. *falciparum* schizont extract (PfSE), or uninfected RBC extract (uE) for 7 days, then the frequency of FoxP3+CD25+CD127^low^ cells among CD4+ T cells was measured. **B.** Ex vivo frequencies of FoxP3+CD25+CD127^low^ regulatory T cells in samples used for stimulation assays. **C.** In PBMCs from malaria-naïve adults, incubation with PfSE resulted in increased frequencies of T_regs_ compared to PBMCs cultured with uE. See also Fig 5A and 5B.
(PDF)

S5 Fig Additional measures of T_reg_ apoptosis. Activated Caspase 3 **(A)** and AnnexinV **(B)** staining of T_regs_ from 28 month old children (PROMOTE, no chemoprevention control arm, with low (<2 episodes ppy) and high (>6 episodes ppy) prior malaria incidence was measured ex vivo and following stimulation with camptothecin (an activator of apoptosis) or *P*. *falciparum* antigen.
(PDF)
