# Supplementary material for: Decline of FoxP3+ Regulatory CD4 T Cells in Peripheral Blood of Children Heavily Exposed to Malaria
Source: PLoS Pathog. 2015 Jul 16;11(7):e1005041. doi: 10.1371/journal.ppat.1005041 (PMC4504515; doi:10.1371/journal.ppat.1005041)
Supplement: S1 Table — (PDF) [file ppat.1005041.s001.pdf]

S1 Table: Cohort characteristics

Name of study cohort	PROMOTE		TCC	PRISM	
	**control**	**SP**		**Nagongera Tororo**	**Walukuba Jinja**
Transmission (aEIR, bites ppy)[#]	High (310)	High (310)	High (310)	High (310)	Low (2.8)
Chemoprevention	X	Monthly SP	X	X	X
Number of children enrolled	82	41	75	91	34
Age years (median, IQR)	2 (2-2)	2 (2-2)	4.3 (4.0-4.3)	6.3 (4.3-8.2)	5.8 (3.6-7.1)
Symptomatic malaria at time of blood draw, n (%)	3 (3.7%)	3 (7.3%)	1 (1.3%)	2 (2.2%)	0
Asymptomatic infection (blood smear) at time of blood draw, n (%)	3 (3.7%)	8 (19.5%)	19 (25.3%)	20 (22%)	0
Clinical characteristics prior to blood draw					
Observation time (median years, IQR)	1.5 (1.5-1.5)	1.5 (1.5-1.5)	3.7 (3.6-3.9)	2.5 (2.5-2.5)	2.0 (2.0-2.0)
Number of children with any episodes of malaria	79 (96.3%)	41 (100%)	73 (97.3%)	91 (100%)	15 (44.1%)
Overall malaria incidence (median, IQR)	5.3 (2.7-7.3)	6.7 (3.0-12.1)	5.7 (3.7-7.1)	3.6 (2.0-4.8)	0 (0-0.5)
Days since last episode of malaria (median IQR)^	27 (11-70)	29.5 (19-61.5)	35 (20-70)	62 (37-187)	230 (201-606)
Clinical characteristics following blood draw					
Observation time (median years, IQR)	1 (1-1)	1 (1-1)	0.77 (0.72-0.95)	0.96 (0.94-0.97)	1 (1-1)
Number of children with any episodes of malaria	78 (95.1%)	40 (97.6%)	67 (89.3%)	83 (91.2%)	1 (2.9%)
Overall malaria incidence (ppy, IQR)	7 (4-11)	10 (4-13)	5.4 (2.6-7.0)	3.2 (1.1-5.2)	0 (0-0)
Days until next episode of malaria (median IQR)^	33 (15-86)	24.5 (10-44)	51 (22-92)	56 (30-83)	234 (NA)

Annual entomological inoculation rate from [25]

***** Samples from PROMOTE-Chemoprevention study were taken at 16, 24, 28 and 36 months of age, as indicated in result sections, clinical characteristics here are for 24 months of age

^ Days since and to next malara episode is for children with a least one episode only
