# Supplementary material for: Decline of FoxP3+ Regulatory CD4 T Cells in Peripheral Blood of Children Heavily Exposed to Malaria
Source: PLoS Pathog. 2015 Jul 16;11(7):e1005041. doi: 10.1371/journal.ppat.1005041 (PMC4504515; doi:10.1371/journal.ppat.1005041)
Supplement: S1 Fig — Frequencies of regulatory T cells were enumerated by staining whole blood samples (2yo cohort) or frozen PBMC samples (4yo cohort). A. For 2yo, whole blood was stained and cells analyzed on four-color Accuri flow-cytometer. B. For 4yo samples, PBMCs were thawed and stained and analyzed on a LSRII. See also Fig 1A and 1B. (PDF) [file ppat.1005041.s002.pdf]

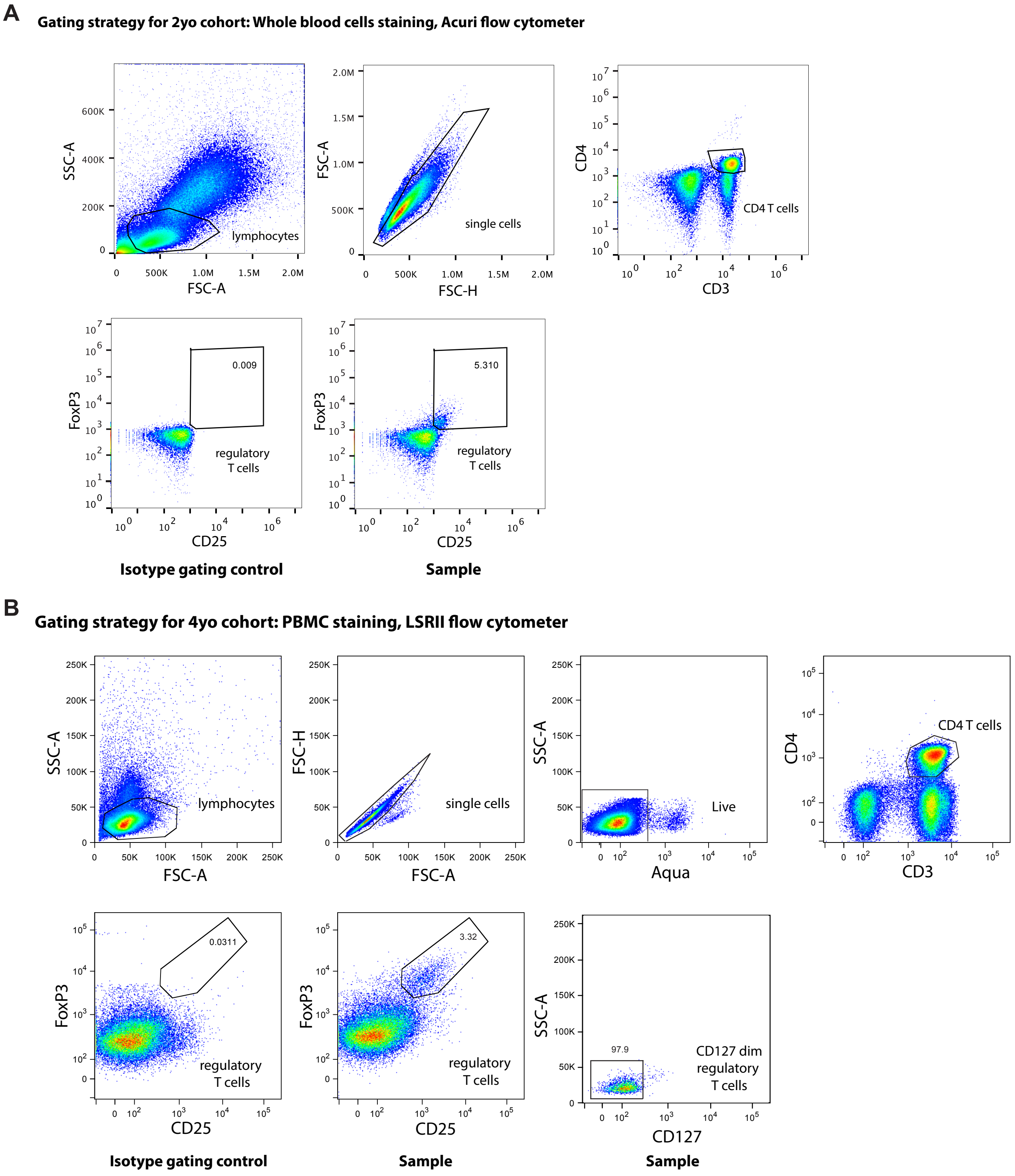

S1 Figure: Gating strategy for regulatory T cells

Frequencies of regulatory T cells were enumerated by staining whole blood samples (2yo cohort) or frozen PBMC samples (4yo cohort). **A.** For 2yo, whole blood was stained and cells analyzed on four-color Accuri flow-cytometer. **B.** For 4yo samples, PBMCs were thawed and stained and analyzed on a LSRII. FoxP3+CD25+ regulatory T cells were further gated on CD127 dim to enumerate regulatory T cells as FoxP3+CD25+CD127dim. This gating strategy was also used for PRISM samples. See also Figure 1a and 1b.
