# Supplementary figures and images for: Decline of FoxP3+ Regulatory CD4 T Cells in Peripheral Blood of Children Heavily Exposed to Malaria
Source: PLoS Pathog. 2015 Jul 16;11(7):e1005041. doi: 10.1371/journal.ppat.1005041 (PMC4504515; doi:10.1371/journal.ppat.1005041)

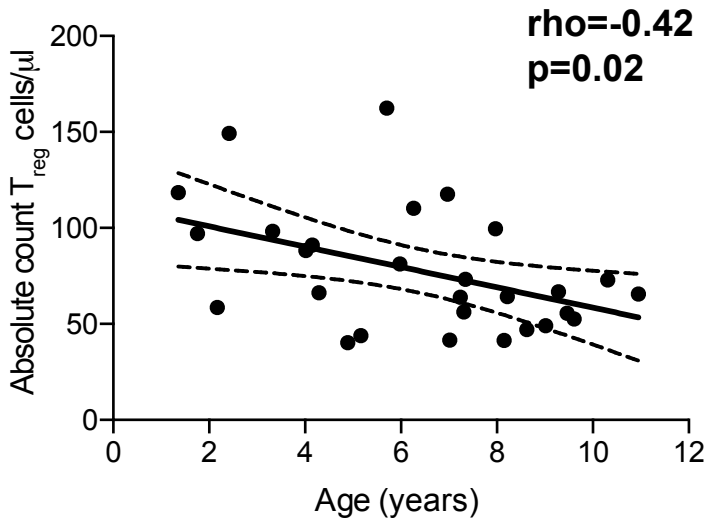

Supplement: S2 Fig — Absolute count of regulatory T cells, analyzed as the percent of FoxP3+CD25+CD127dim expressing CD4+ T cells, normalized to CD4+ T cell absolute counts, from 1 to 11 year old children (PRISM cohort, high transmission Nagongera, Tororo District), declined with increasing age. (PDF) [file ppat.1005041.s003.pdf]

**A**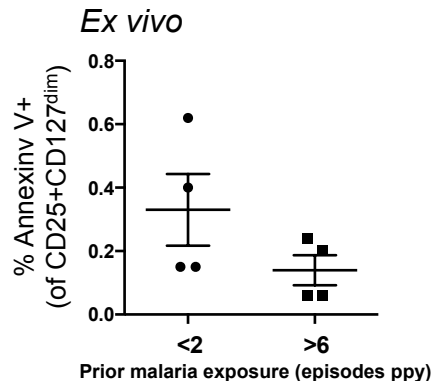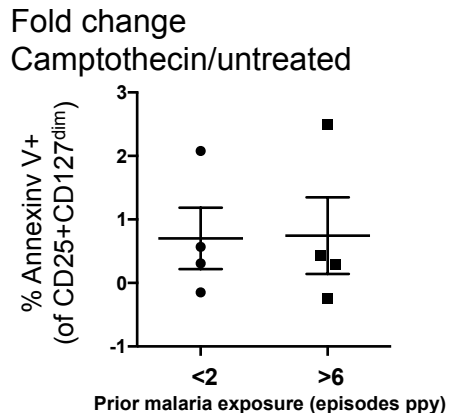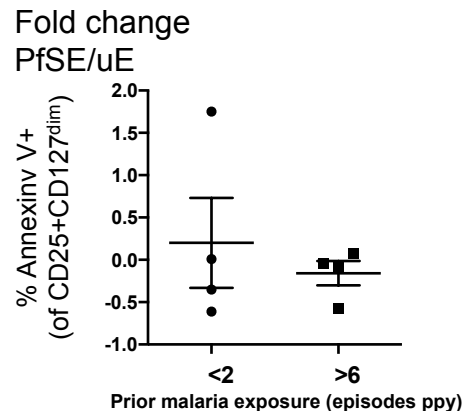**B**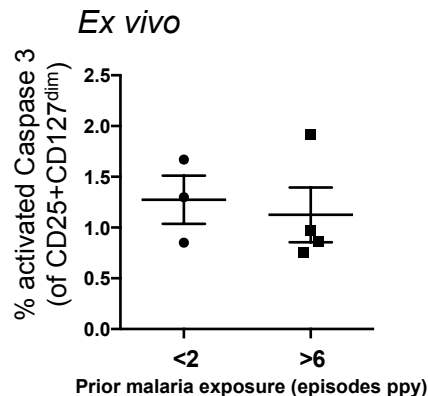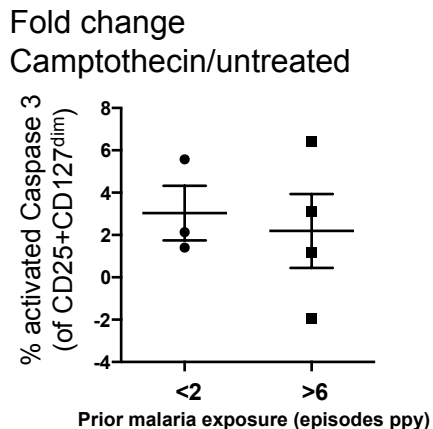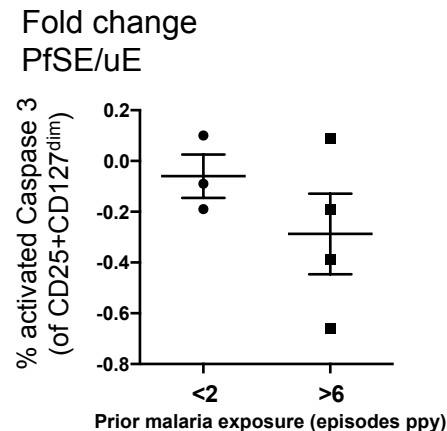

Supplement: S5 Fig — Activated Caspase 3 (A) and AnnexinV (B) staining of Tregs from 28 month old children (PROMOTE, no chemoprevention control arm, with low (<2 episodes ppy) and high (>6 episodes ppy) prior malaria incidence was measured ex vivo and following stimulation with camptothecin (an activator of apoptosis) or P. falciparum antigen. (PDF) [file ppat.1005041.s006.pdf]
