# Supplementary material for: Decline of FoxP3+ Regulatory CD4 T Cells in Peripheral Blood of Children Heavily Exposed to Malaria
Source: PLoS Pathog. 2015 Jul 16;11(7):e1005041. doi: 10.1371/journal.ppat.1005041 (PMC4504515; doi:10.1371/journal.ppat.1005041)
Supplement: S3 Fig — Frequencies of regulatory T cells expression TNFR2 were enumerated by staining PBMCs. Gating for FoxP3+CD25+CD127dim regulatory T cells was as for S1 Fig. TNFR2 staining was gated on FMO controls, as indicated. (PDF) [file ppat.1005041.s004.pdf]

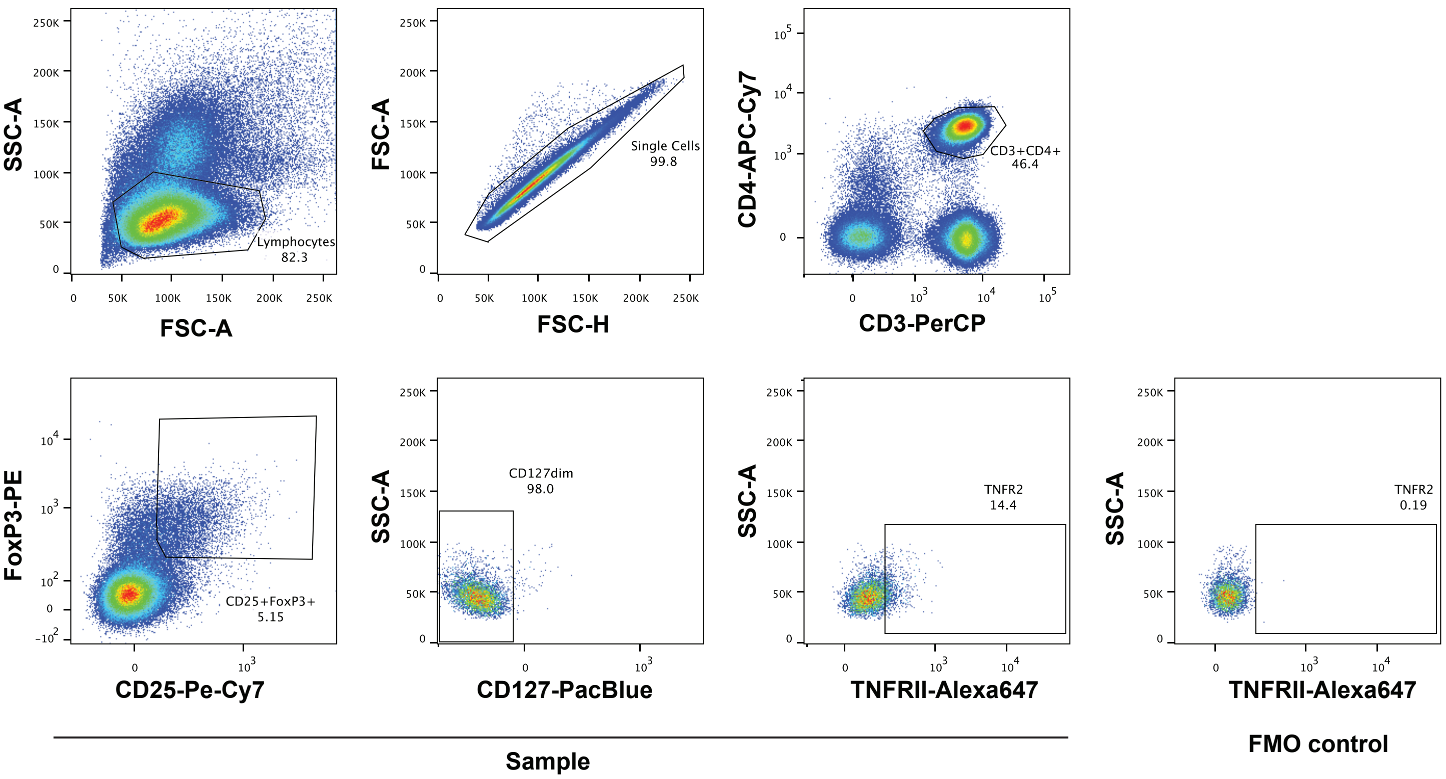

S3 Figure: Gating strategy for TNFRII expression

Frequencies of regulatory T cells expression TNFR2 were enumerated by staining PBMCs. Gating for FoxP3+CD25+CD127dim regulatory T cells was as for S1 Figure. TNFR2 staining was gated on FMO controls, as indicated.
