# Supplementary material for: Decline of FoxP3+ Regulatory CD4 T Cells in Peripheral Blood of Children Heavily Exposed to Malaria
Source: PLoS Pathog. 2015 Jul 16;11(7):e1005041. doi: 10.1371/journal.ppat.1005041 (PMC4504515; doi:10.1371/journal.ppat.1005041)
Supplement: S4 Fig — A. PBMCs were incubated with P. falciparum schizont extract (PfSE), or uninfected RBC extract (uE) for 7 days, then the frequency of FoxP3+CD25+CD127low cells among CD4+ T cells was measured. B. Ex vivo frequencies of FoxP3+CD25+CD127low regulatory T cells in samples used for stimulation assays. C. In PBMCs from malaria-naïve adults, incubation with PfSE resulted in increased frequencies of Tregs compared to PBMCs cultured with uE. See also Fig 5A and 5B. (PDF) [file ppat.1005041.s005.pdf]

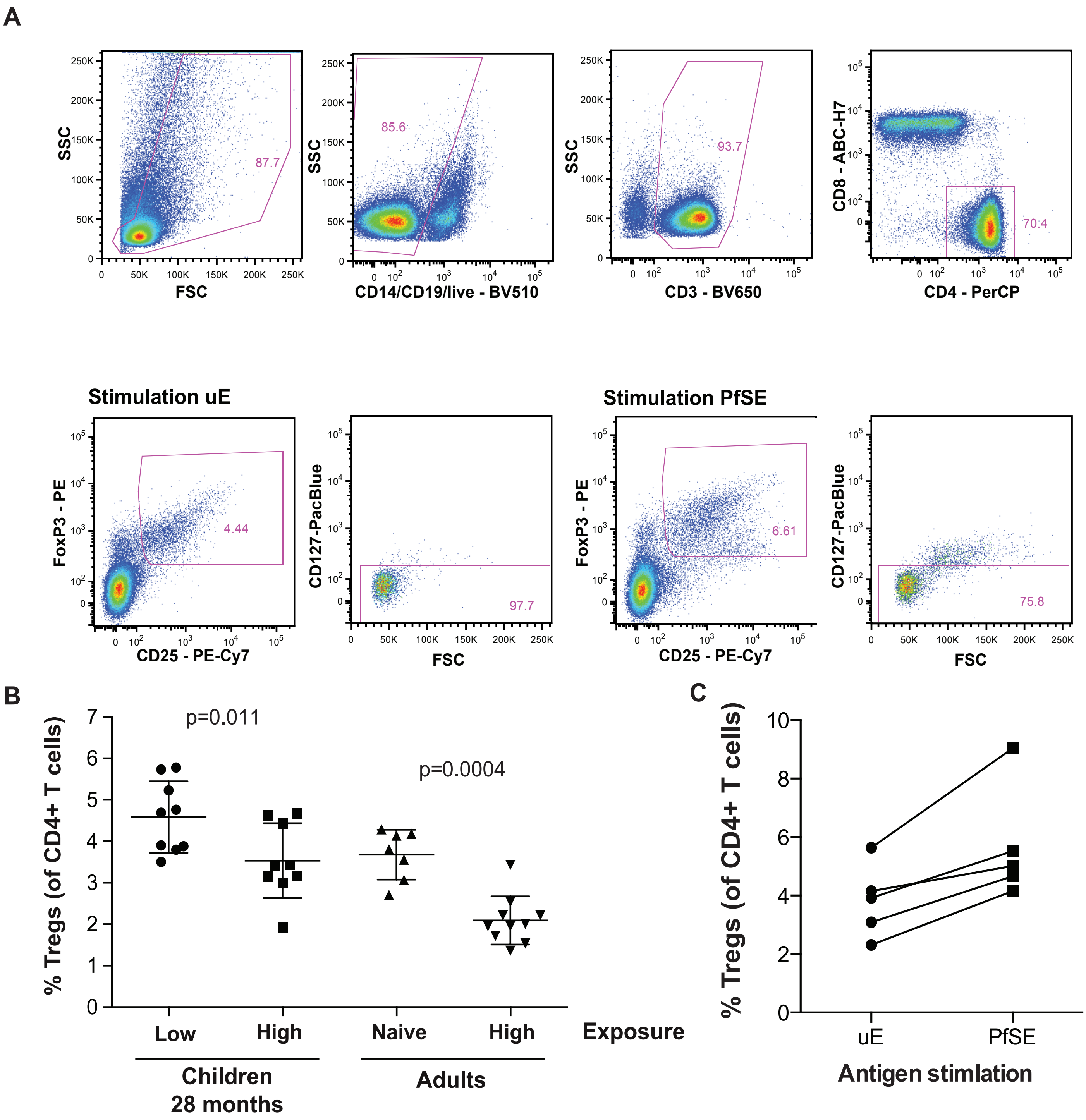

S4 Figure: Regulatory T cell induction assays

A. PBMCs were incubated with *P. falciparum* schizont extract (PfSE), or uninfected RBC extract (uE) for 7 days, then the frequency of FoxP3+CD25+CD127^{low} cells among CD4+ T cells was measured. **B.** *Ex vivo* frequencies of FoxP3+CD25+CD127^{low} regulatory T cells in samples used for stimulation assays. **C.** In PBMCs from malaria-naïve adults, incubation with PfSE resulted in increased frequencies of T$_{regs}$ compared to PBMCs cultured with uE. See also Figure 5a and 5b.
